# Supplementary material for: A Theoretical Study of Fe Adsorbed on Pure and Nonmetal (N, F, P, S, Cl)-Doped Ti3C2O2 for Electrocatalytic Nitrogen Reduction
Source: Nanomaterials (Basel). 2022 Mar 25;12(7):1081. doi: 10.3390/nano12071081 (PMC9000748; doi:10.3390/nano12071081)
Supplement: Supplementary file 1 [file nanomaterials-12-01081-s001.zip › nanomaterials-1643925-supplementary.pdf]

nanomaterials

Supporting Information

A Theoretical Study of Fe Adsorbed on Pure and Nonmetal (N, F, P, S, Cl)-Doped $Ti_3C_2O_2$ for Electrocatalytic Nitrogen Reduction

Heng Luo [1,2], Xiaoxu Wang [3], Chubin Wan [2,*], Lu Xie [1], Minhui Song [1] and Ping Qian [1,2]

1 Beijing Advanced Innovation Center for Materials Genome Engineering, University of Science and Technology Beijing, Beijing 100083, China; s20190798@xs.ustb.edu.cn (H.L.); b20170555@xs.ustb.edu.cn (L.X.); s20191385@xs.ustb.edu.cn (M.S.); qianping@ustb.edu.cn (P.Q).
2 Department of Physics, University of Science and Technology Beijing, Beijing 100083, China
3 DP Technology, Beijing 100083, China; wangxx@dp.tech
* Correspondence: cbwan@ustb.edu.cn

Supporting Information:

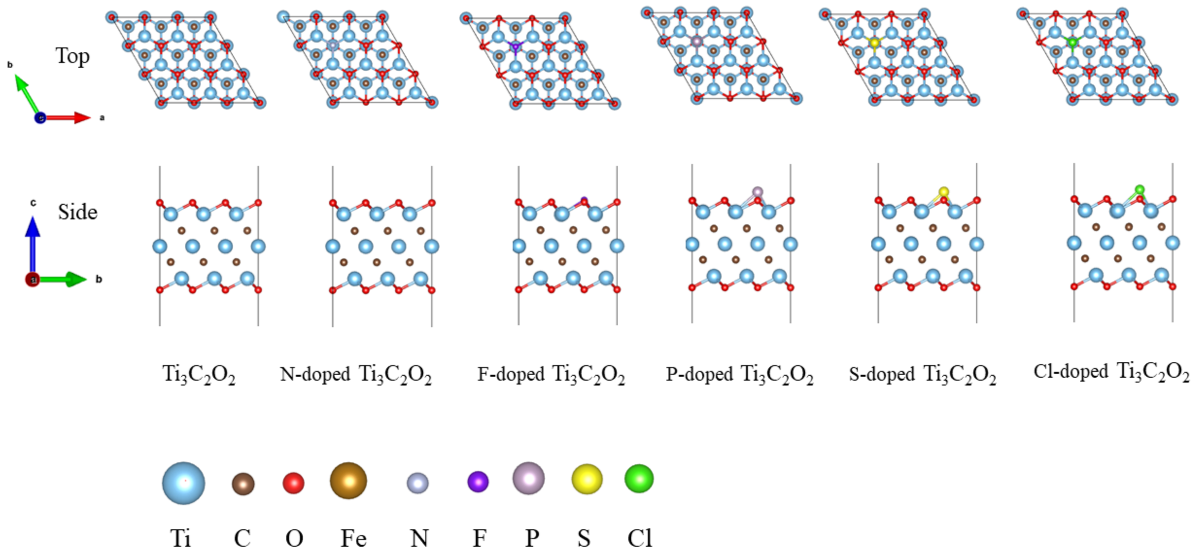

Figure S1. Top and side views of $Ti_3C_2O_2$ and its nonmetal doped structure.

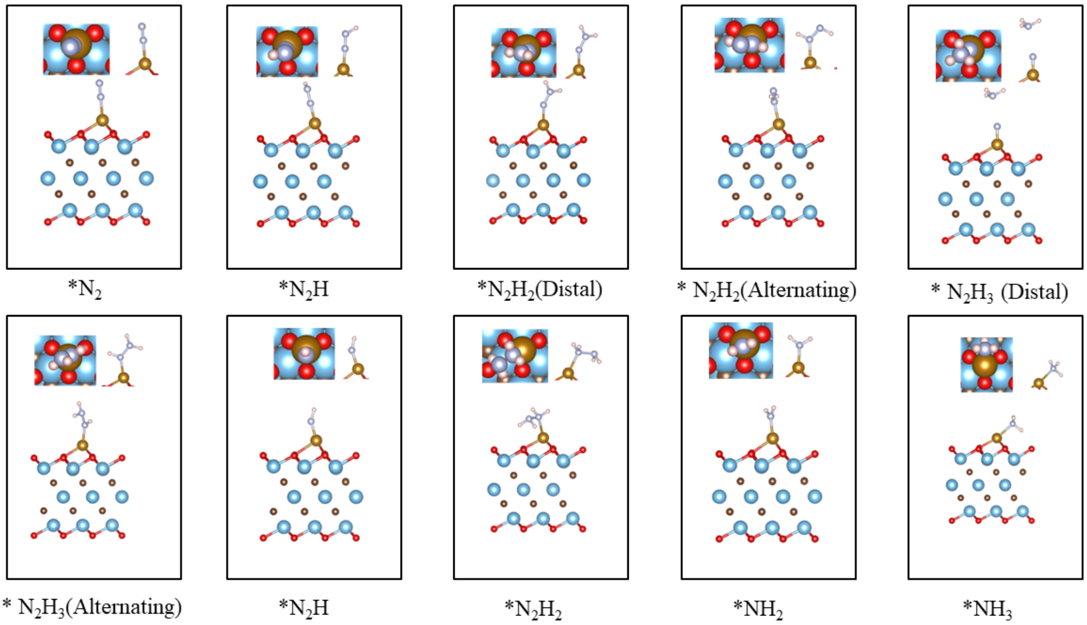

Figure S2. Optimized structures of all the possible elementary steps in NRR, taking $Ti_3C_2O_2$ as an example. Other nonmetal-doped $Ti_3C_2O_2$ show similar geometric structures.
